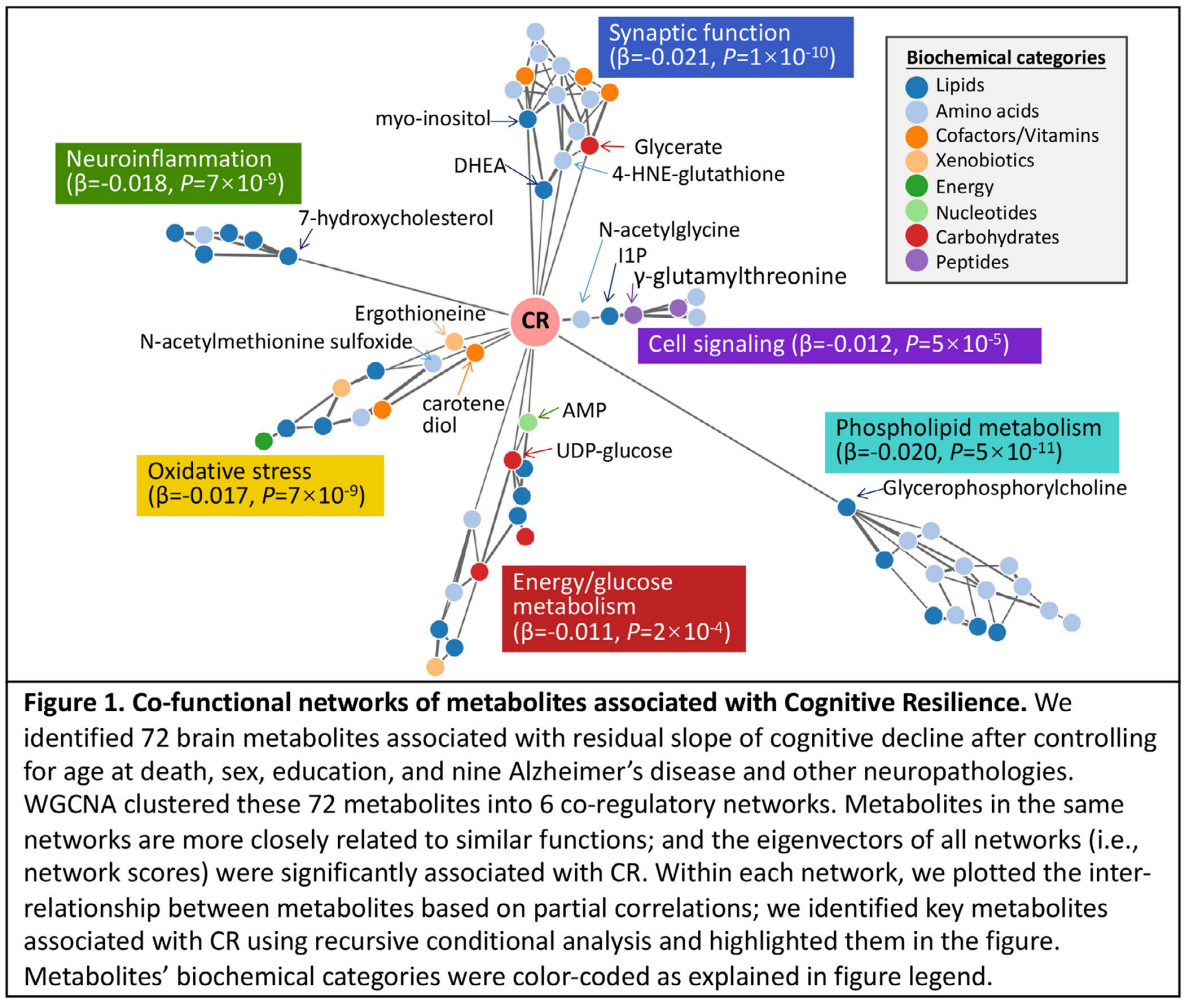# Brain metabolic alterations associated with cognitive resilience

**DOI:** 10.1002/alz.089181

**Published:** 2025-01-09

**Authors:** Jun Li, Ana W. Capuano, David A. Bennett, Liming Liang, Zoe Arvanitakis, Francine Grodstein

**Affiliations:** ^1^ Harvard T.H. Chan School of Public health, Boston, MA USA; ^2^ Brigham and Women's Hospital and Harvard Medical School, Boston, MA USA; ^3^ Rush University Medical Center, Chicago, IL USA; ^4^ Rush Alzheimer's Disease Center, Chicago, IL USA; ^5^ Rush Alzheimer's Disease Center, Rush University Medical Center, Chicago, IL USA; ^6^ Rush University, Chicago, IL USA

## Abstract

**Background:**

Cognitive resilience (CR) refers to the continuum from worse to better‐than‐expected cognition, given the degree of neuropathology. Understanding mechanisms underlying CR could inform discovery of novel targets for dementia prevention; however, specific metabolic pathways underlying CR are yet to be elucidated.

**Methods:**

Our study included 484 deceased participants (mean age at death =91 years, 70.2% women) from the Religious Orders Study and Rush Memory and Aging Project. Metabolomics profiling was conducted at Metabolon Inc. by a chromatography–mass spectrometry in postmortem dorsolateral prefrontal cortex tissue, yielding 600 known metabolites quantified for analysis. Antemortem cognitive function was assessed annually by 17 neuropsychologic tests through death, and neuropathologies were evaluated in postmortem brain. CR was defined as the residual slope of cognitive decline after controlling for age at death, sex, education, and nine Alzheimer’s disease, other neurodegenerative, and cerebrovascular neuropathologies. We analyzed the associations between individual brain metabolites and CR score using linear regressions. We also constructed co‐regulatory networks for metabolites significantly related to CR, and identified key metabolites for CR within each network using recursive conditional analysis.

**Results:**

We identified 72 metabolites whose levels in the prefrontal cortex were associated with CR (FDR<0.05), encompassing diverse biochemical categories including 33 amino acids and peptides, 21 lipids, 7 cofactors and vitamins, 6 carbohydrates/energy metabolites, and 5 others. Based on partial correlations, the 72 CR‐related metabolites were constructed into 6 co‐regulatory networks, each linked to specific biological functions including oxidative stress (key metabolites driving the associations were N‐acetylmethionine sulfoxide, ergothioneine, and carotene diol), synaptic function (led by myo‐inositol, glycerate, and docosahexaenoyl ethanolamide), neuroinflammation (led by 7‐hydroxycholesterol), cell signaling (led by inositol 1‐phosphate and gamma‐glutamylthreonine), phospholipid metabolism (led by glycerophosphorylcholine), and energy/glucose metabolism (led by UDP‐glucose and adenosine monophosphate). The eigenvectors of all 6 co‐functional networks were significantly associated with CR (FDR<0.05).

**Conclusion:**

We identified brain metabolites implicated in oxidative stress and necroinflammation, synaptic function and cell signaling, and lipid/energy metabolism, associated with rates of cognitive decline after controlling for neuropathologies. These CR‐related metabolites may inform the discovery of novel targets for improving cognitive resilience to neuropathology.